# Factors predicting outcome in whiplash injury: a systematic meta-review of prognostic factors

**DOI:** 10.1007/s10195-016-0431-x

**Published:** 2016-10-13

**Authors:** Pooria Sarrami, Elizabeth Armstrong, Justine M. Naylor, Ian A. Harris

**Affiliations:** 1 Institute of Trauma and Injury Management, New South Wales Agency for Clinical Innovation, Level 4, Sage Building, 67 Albert Avenue, Chatwswood, Sydney, NSW 2067 Australia; 2 South Western Sydney Clinical School, UNSW, Sydney, Australia; 3 Neuroscience Research Australia, Sydney, Australia; 4 South Western Sydney Local Health District, Liverpool Hospital, Liverpool, Australia

**Keywords:** Whiplash injury, Acute whiplash injury, Motor vehicle accidents, Prognosis, Prognostic factors, Risk factors, Outcome prediction, Meta-review, Psycho-social factors, Physical factors

## Abstract

**Background:**

Whiplash injuries are among the leading injuries related to car crashes and it is important to determine the prognostic factors that predict the outcome of patients with these injuries. This meta-review aims to identify factors that are associated with outcome after acute whiplash injury.

**Materials and methods:**

A systematic search for all systematic reviews on outcome prediction of acute whiplash injury was conducted across several electronic databases. The search was limited to publications in English, and there were no geographical or time of publication restrictions. Quality appraisal was conducted with A Measurement Tool to Assess Systematic Reviews.

**Results:**

The initial search yielded 207 abstracts; of these, 195 were subsequently excluded by topic or method. Twelve systematic reviews with moderate quality were subsequently included in the analysis. Post-injury pain and disability, whiplash grades, cold hyperalgesia, post-injury anxiety, catastrophizing, compensation and legal factors, and early healthcare use were associated with continuation of pain and disability in patients with whiplash injury. Post-injury magnetic resonance imaging or radiographic findings, motor dysfunctions, or factors related to the collision were not associated with continuation of pain and disability in patients with whiplash injury. Evidence on demographic and three psychological factors and prior pain was conflicting, and there is a shortage of evidence related to the significance of genetic factors.

**Conclusions:**

This meta-review suggests an association between initial pain and anxiety and the outcome of acute whiplash injury, and less evidence for an association with physical factors.

**Level of evidence:**

Level 1.

**Electronic supplementary material:**

The online version of this article (doi:10.1007/s10195-016-0431-x) contains supplementary material, which is available to authorized users.

## Introduction

Whiplash injury, or whiplash-associated disorder, can be defined as a collection of neck-related symptoms following a car accident [1] and is among the leading car crash-related injuries with respect to burden on patients, the healthcare system and insurance organisations. The incidence of whiplash injury has been increasing during the past decades [2], ranging from 16 to 200 per 100,000 population, and varying by geographical location [3]. In addition, patterns of crashes causing whiplash injury are changing, now including minor accidents of any type [4]. The increasing incidence may also be due to the rise in traffic density, and changes in societal and litigation factors [5]. It is estimated that 50 % of patients with acute whiplash injury develop long-term disability [6].

**Fig. 1:**
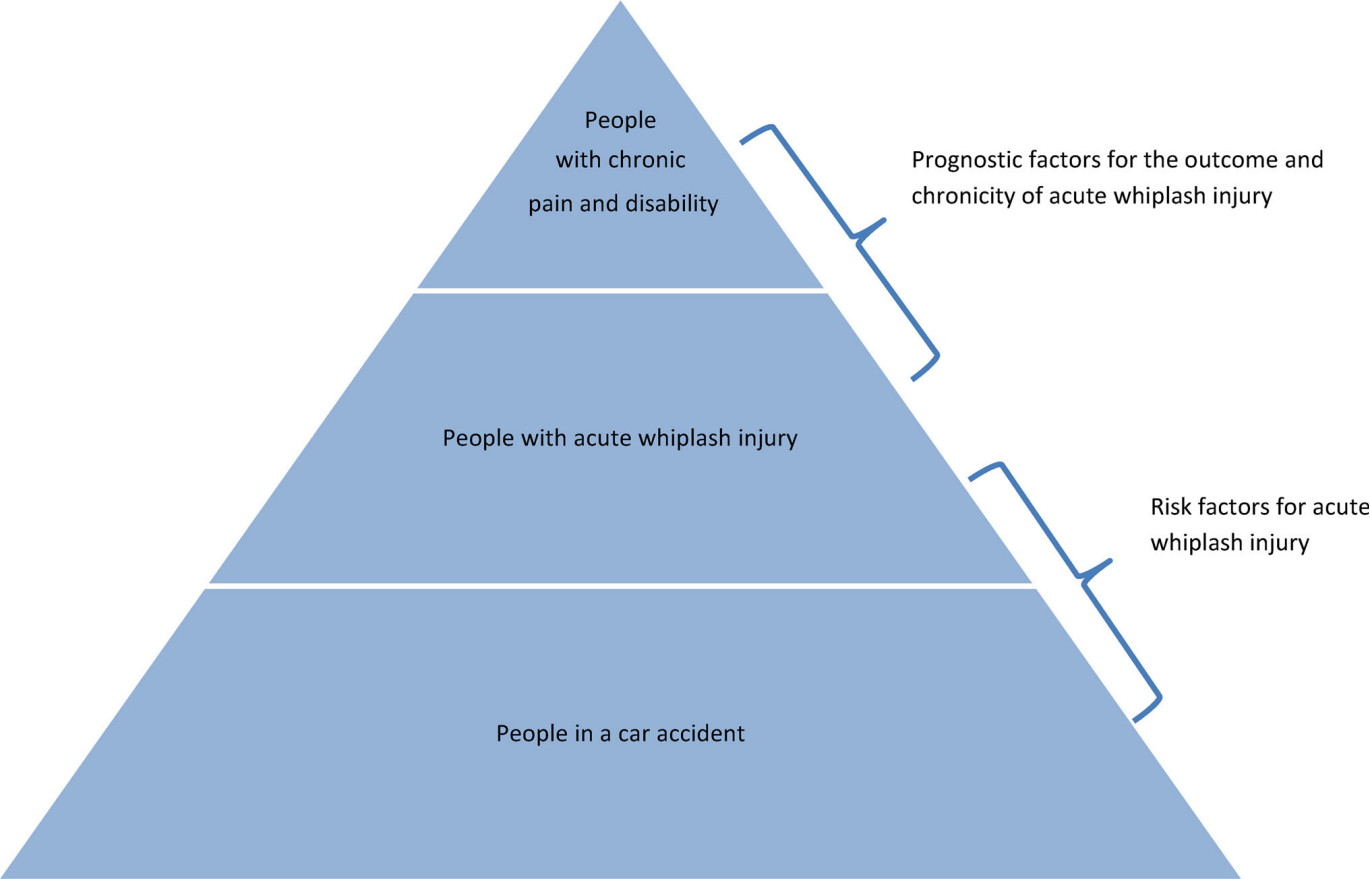
Illustration of risk factors and prognostic factors of acute whiplash injury

While various factors are considered to be related to the incidence and chronicity of acute whiplash injury, it is important to distinguish between risk factors for acute whiplash and prognostic factors for a poor outcome and chronicity in people who have sustained an acute whiplash injury (Fig. 1) [7]. Walton et al. have undertaken an overview of systematic reviews on prognostic factors in neck pain and have suggested that baseline neck pain intensity and disability are strongly associated with outcome, while trauma-related parameters have no effect on outcome [8]. Nevertheless, Walton et al. suggested the need for further work in this area. Considering the availability of more recent systematic reviews on the topic, we have undertaken a more focused systematic meta-review on the prognostic factors of outcome after acute whiplash injury, which aimed to answer the following questions: what is the quality of currently available systematic reviews on the prediction of outcome after acute whiplash injury; and which factors predict outcome after acute whiplash injury?

## Materials and methods

As our preliminary search found several relevant systematic reviews, it was deemed feasible to undertake a meta-review [9]. A meta-review is a systematic overview of reviews, in which all available systematic reviews are included and rigorous appraisal of each included systematic review is undertaken [9]. Since each paper included in this study is a systematic review that has appraised a number of studies, this study has the opportunity to present a comprehensive and reliable picture of the field. The PRISMA statement guided the approach [10] (S1 PRISMA Checklist).

To identify the relevant papers, the medical subject heading (MeSH) of ‘whiplash’ and an extensive list of MeSH subheadings and a combination of relevant phrases were used (S2 Table 5). The lists of MeSH subheadings varied according to differences in the various databases. However, to ensure the sample would be a comprehensive collection of relevant systematic reviews, an attempt was made to over-include MeSH subheadings (i.e., subheadings that were not directly related to prognostic factors were also included). The electronic databases searched were: PubMed, Medline, Embase, Cochrane library, CINAHL and PsycINFO. The search was limited to publications in English, but was not limited by date of publication or geographical location. Non-systematic reviews, opinions, books, book chapters, discussions and letters were excluded. Other meta-reviews were cited and compared with this study, but not included in data analysis.

During the screening phase, we included systematic reviews if they directly reported results on whiplash and we excluded reviews if they combined data related to whiplash with other musculoskeletal injuries. We also included only systematic reviews that explored prognostic factors, as outlined in the background section, and excluded papers that explored other issues such as the determinants of incidence of acute whiplash injury. Studies were considered as systematic reviews if they clearly introduced the searched databases and key terms, and reported the number of identified papers. Papers were first screened for their topic and methodology based on their titles and abstracts. The full texts of selected papers were then obtained, and evaluated independently by two reviewers (PS and EA). The results of the two reviewers were compared, and any disagreements were resolved by discussion.

After including a number of systematic reviews based on their topic and methodology, the quality of the included systematic reviews was assessed using A Measurement Tool to Assess Systematic Reviews (AMSTAR) [11].

Data analysis involved producing a list of prognostic factors for each systematic review, and then the conclusions obtained from each systematic review were recorded for each factor.

The conclusion of each review for each identified prognostic factor was determined and recorded using the following classification: (1) associated: when the systematic review found adequate evidence to conclude that a prognostic factor was associated with the outcome of acute whiplash injury; (2) non-associated: when the systematic review found adequate evidence to conclude that a prognostic factor was not associated with the outcome of whiplash; (3) lack of evidence: when the systemic review reported being unable to identify adequate evidence regarding a prognostic factor; and (4) controversial: when the systematic review found controversial or conflicting evidence regarding a prognostic factor.

A prognostic factor was allocated to one of the first three categories (associated, non-associated, or lack of evidence) whenever the majority of the systematic reviews that analysed each factor agreed on the association or lack of association with the outcome, or if they referred to a lack of evidence. A prognostic factor was placed in the fourth category (controversial) if the majority of the systematic reviews referred to controversial evidence, or if we identified controversial conclusions in the systematic reviews.

A priori, the intent of the analysis was to indicate the overall direction of current evidence for each of the prognostic factors in a qualitative manner with no report on quantitative strength of effects.

## Results

**Fig. 2:**
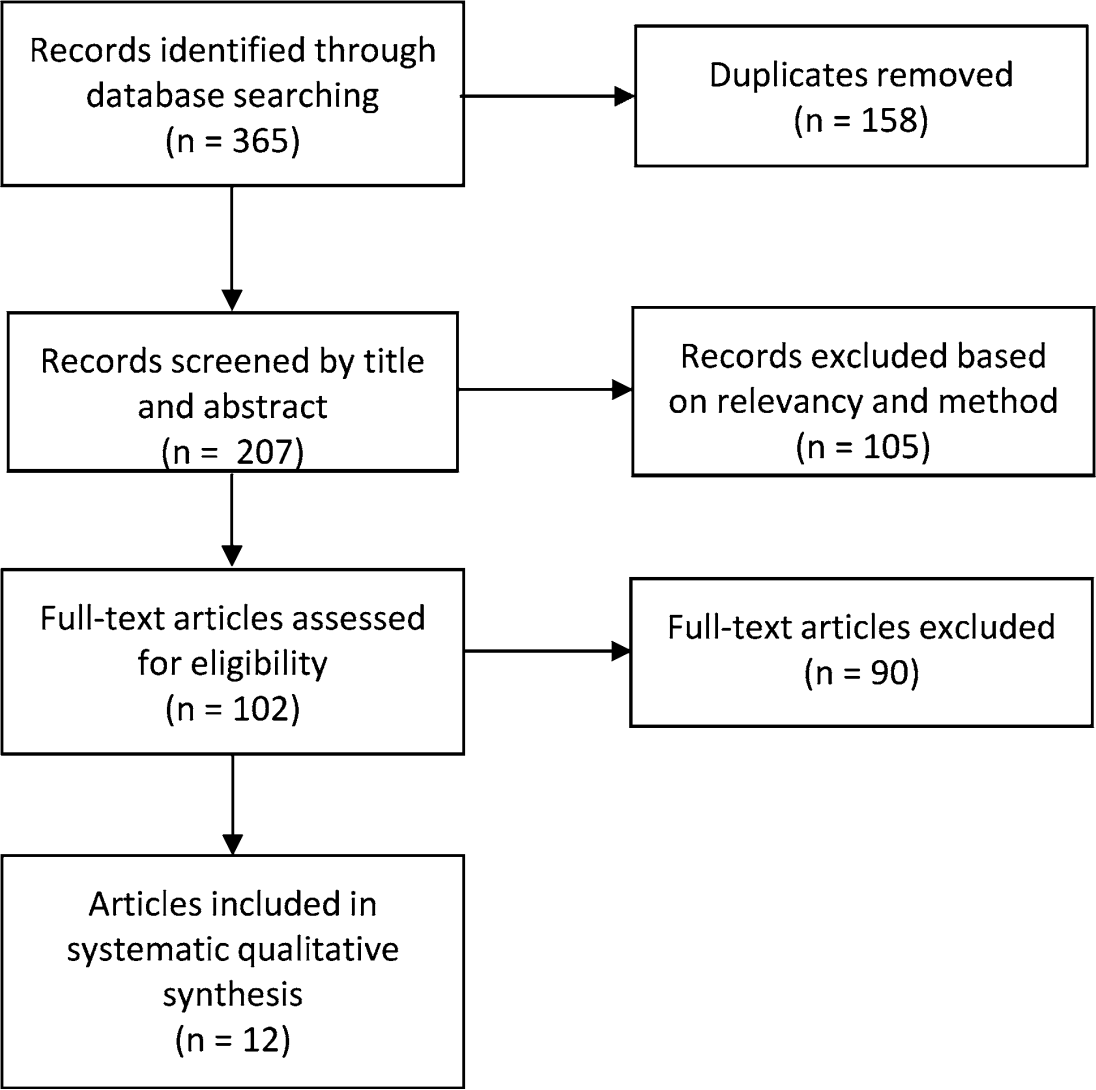
Summary of the screening process

Initial searches in different databases yielded 365 articles, and the screening process for these articles is summarised in Fig. 2. Of the 365 articles found, 158 were duplicates, 105 items were excluded based on the evaluation of title and abstract and 90 papers were excluded after appraisal of their full text (S3 Table 6. Excluded studies). The remaining 12 papers (referenced in Table 1) were rated for quality using the AMSTAR tool as moderate quality (score 5–8) and their average score was 6.7 (out of 11, with the range of 6–8). They included systematic reviews focussing on whiplash injuries with no fractures or dislocations.

**Table 1:** Systematic reviews included

References	Years of reviewed literature	Aim of the review	Databases searched	Number of studies included	Total number of patients in the included studies
Li et al. 2013 [12]	Not mentioned	To evaluate the relationship of MRI signal changes of alar and transverse ligaments and whiplash-associated disorders	PubMed, Embase, Cochrane Library	6	622
Daenen et al. 2013 [13]	To October 2011	To explore cervical motor dysfunctions in acute whiplash-associated disorders/to evaluate their course and assess their predictive value for long-term recovery	PubMed and Web of Science	10	1416
Walton et al. 2009 [14] and 2013 [15]	To May 2007 (then to May 2012)	To assess risk factors for persistent problems following whiplash after a motor vehicle accident	MEDLINE, CINAHL, PsycINFO, Embase	11 (then adding 4)	3193 (then adding 1121)
Spearing et al. 2012 [16]	To April 2010	To examine the evidence on the compensation hypothesis in relation to compensatable whiplash injuries	MEDLINE/PubMed, CINAHL, Embase, PEDro, PsycINFO, CCTR, Lexis, EconLit	11	4218
Goldsmith et al. 2012 [17]	To September 2011	To assess whether cold hyperalgesia is a prognostic factor for long-term pain or disability in acute whiplash injury	PubMed-MEDLINE, OVID-MEDLINE, OVID-Embase, OVID-PsycINFO	6	443
Carroll et al. 2008 [18]	1980–2006	To gather the best evidence on neck pain and associated disorders	MEDLINE	29	28,045
Kamper et al. 2008 [19]	To April 2007	To outline the course of recovery, pain and disability symptoms/to evaluate the influence of different prognostic factors on outcome	MEDLINE, Cochrane Database of Systematic Reviews, ACP Journal club, DARE, PsycINFO, Embase	38	19,906
Williamson et al. 2008 [20]	To August 2006	To review the prognostic value of psychological factors in the development of late whiplash syndrome	PubMed, MEDLINE, CINAHL, Embase and PsycINFO	17	2148
Williams et al. 2007 [21]	To August 2006	To review evidence concerning physical prognostic factors for development of late whiplash syndrome	PubMed, MEDLINE, CINAHL, Embase, PsycINFO	26	4261
Scholten-Peeters et al. 2003 [22]	To April 2002	To evaluate prognostic factors associated with functional recovery of patients with whiplash injuries	MEDLINE, Embase, CINAHL, the database of the Dutch Institute of Allied Health Professions	29	18,340
Cote et al. 2001 [23]	1995–2000	To review prognosis of whiplash	MEDLINE (1966 to September 2000), CINAHL (1982 to July 2000), Embase (1980 to January 1999), and Healthstar (1975 to September 2000).	13	15,822

### Prognostic factors

A broad range of prognostic factors was explored by the systematic reviews included. Analysis of the final 12 reviews indicated that four groups of factors were associated with the outcome of acute whiplash injury (Table 2), three groups of factors were identified as non-associated (Table 3), and the evidence was controversial or insufficient for five other factors (Table 4). Heterogeneity and variations in the systematic reviews included precluded quantitative analysis.

**Table 2:** Associated factors

Factors	The conclusion of evaluated systematic reviews [and citations]	Overall
Post-injury pain and disability, whiplash grades, cold hyperalgesia	A [15], A [17], A [18], A [19], A [21], A [22], A [23]	Associated
Post-injury anxiety	A [18], A [20]	Associated (based on outdated reviews)^a^
Catastrophizing	A [18], A [14], C [20]	Associated (based on outdated reviews)
Compensation and legal factors	A [16], A [18], L [23]	Associated
Early healthcare use	A [18], L [23]	Associated (based on outdated reviews)^a^

*A* associated, *L* lack of evidence

^a^Systematic reviews that were published 5 years ago or earlier are considered ‘outdated’

**Table 3:** Non-associated factors

Factors	The conclusion of evaluated systematic reviews [and citations]	Overall
Post-injury MRI or radiological findings	N [12], N [18]	Not associated
Motor dysfunctions	N [13]	Not associated
Collision factors	N [15], N [19], N [18], N [22], C [23]	Not associated

*N* non-associated, *C* controversial

**Table 4:** Factors that were controversial or lacked evidence

Factors	The conclusion of evaluated systematic reviews [and citations]	Overall
Gender	A [15], C [18], N [19], N [22], A [23]	Controversial
Age	N [15], N [19], C [18], N [22], A [23]	Controversial
Education	A [15], C (18], C [23)	Controversial
Pain prior to accident	A [15], C [18], C [23]	Controversial
Genetic factors	L [18]	Lack of evidence
Coping behaviour	C [18], C [20]	Controversial (based on outdated reviews)^a^
General psychological distress	A [19], N [20]	Controversial (based on outdated reviews)^a^
Depressive mood	N [14], A [18], C [20]	Controversial (based on outdated reviews)^a^

*A* associated, *N* non-associated, *C* controversial, *L* lack of evidence

^a^Systematic reviews that were published 5 years ago or earlier are considered ‘outdated’

### Associated factors

Factors associated with the prognosis for people with whiplash injury were (Table 2):

- Post-injury pain and disability (i.e., pain and disability that whiplash patients experience after a car accident), whiplash grades, cold hyperalgesia
- Post-injury anxiety
- Catastrophizing
- Compensation and legal factors
- Early use of healthcare

The most consistent finding of the systematic reviews was the association of post-injury pain and disability with long-term pain and disability. Whether directly exploring this factor, or referring to whiplash grades and cold hyperalgesia, six different systematic reviews suggested the association [15, 17–19, 21, 23]. However, the association of other factors with the prognosis for patients with whiplash is not as strong, although the association of psychosocial factors with a whiplash prognosis is notable. Psychosocial factors are the combination of social factors, for example compensation and legal matters, with psychological factors, such as post-injury anxiety and catastrophizing. As indicated in Table 2, we identified two or three systematic reviews for each of the other factors; some of the available reviews were based on systematic reviews conducted more than 5 years ago, and there were two reviews that reported lack of evidence for some of these factors.

### Non-associated factors

Factors identified as not being associated with the prognosis of whiplash were post-injury magnetic resonance imaging (MRI) or radiological findings; motor dysfunctions; and collision factors (i.e., factors related to the car accident such as the direction of impact, the use of seatbelts or headrests, and the speed of the car at the time of impact [18]). As indicated in Table 3, the lack of association of collision factors with the prognosis of whiplash was confirmed based on four reviews, while we identified only one or two reviews covering each of the other two factors. It is notable that the list of non-associated factors is more related to ‘physical’ and biological items.

### Controversial or insufficient evidence

Current evidence is conflicting for the association of demographic factors (gender, age and education), three psychological factors (coping behaviour, general psychological distress and depressive mood) and pre-accident pain with the prognosis of whiplash. A lack of evidence is reported for genetic factors.

## Discussion

This meta-review, summarising the results of twelve systematic reviews, indicates that the outcome of patients with acute whiplash injury is associated with post-injury symptoms and some psychosocial factors, and not injury-related physical or mechanical factors. These findings are consistent with a previous meta-review that explored prognostic factors of neck pain in general [8]. To summarise and simplify the result of this meta-review, a ‘typical’ whiplash patient with a poor outcome (that is, prolonged pain and disability) can be depicted as having severe pain and anxiety, and is seeking or has sought legal advice and early healthcare use. The type of accident, findings on physical examination, or radiological investigations will not affect the prognosis. Thus, a patient suffering chronic pain and disability post-whiplash can potentially be involved in a minor car accident with no motor dysfunction or radiological abnormality. The association of some psychosocial factors with the chronicity of whiplash injury is in accordance with previous studies involving chronic pain patients, which indicate a similar association between psychosocial factors and the course of chronic pain in general [24], and other forms of chronic pain such as non-specific low back pain [25, 26].

It is also notable that current evidence is conflicting or lacking on factors such as demographic factors (age, gender and education), three psychological factors and pain prior to accident. It is notable that Walton et al. concluded in their meta-review, with moderate confidence, that age has no effect on the outcome of whiplash [8]. This contrasts with our analysis, which concluded controversial evidence based on an association reported by Cote et al. [23]. This lack of conclusiveness might be explained by differences in the methodologies of various studies, such as different sample frames (normal population, insurance population or hospital emergency departments) [13, 16, 23]. In addition, the effect of demographic factors is not usually direct, but is mediated by other factors [27]; therefore, future studies should consider the role of confounding factors, such as comorbid mental health problems, while exploring the association of demographic factors with the prognosis of whiplash injury.

All twelve papers included in this review emphasised the need for more rigorous evidence, and made suggestions for future work in this field. These included the need for further studies on some of the prognostic factors, the need to explore the causal effect of other factors, and studies assessing the possibility of using prognostic factors in the prevention or treatment of whiplash whenever possible, as discussed below.

Carroll et al. reported a lack of high-quality studies on the association of the following items with the prognosis of whiplash: occupation type, disc degeneration, cultural factors, pre-injury fitness or exercise, and pre-existing or new incidence of widespread body pain or fibromyalgia [6]. Cote et al. emphasised that, based on current evidence, it is not clear whether the course of whiplash differs in patients recruited from the general population compared to those recruited from emergency departments or primary care practice [23]. Spearing et al. could not find any studies that directly explored the role of receiving compensation payment on the prognosis of whiplash patients [16]. Finally, Williamson et al. reported a lack of high-quality evidence on the association of psychological factors and chronicity of acute whiplash injury [20]. These areas should be investigated in any future studies.

The association of a factor with the prognosis of whiplash does not necessarily reflect a causal relationship; such associated factors cannot therefore be necessarily used as a basis for the treatment or prevention of whiplash. More studies are necessary to investigate the potential role of prognostic factors on aetiology, prevention and treatment of whiplash. For example, although cold hyperalgesia is associated with pain and disability in whiplash patients, more studies are needed to investigate whether cold hyperalgesia can be considered as a cause of pain, or if there are other confounding factors [28]. Another example is related to the role of compensation, which is associated with poor health outcome [29, 30]; however, studies have yet to explore reverse causality, that is, the poor outcome being the cause of compensation-seeking [16, 31].

In addition, future studies should explore whether a patient’s outcome can be improved by removing a prognostic factor. For example, while whiplash patients who report back pain following a car accident are more likely to have a poor outcome, more studies are needed to determine if treating the back pain can improve the outcome of whiplash [15].

Considering the complexities that exist around the association of factors with outcome of a health condition such as acute whiplash injury, complete elaboration of such associations would be beyond the scope of a single study, and different phases of research might be needed to identify, confirm and understand prognostic associations [32]. It is also necessary that future studies employ rigorous methodology (such as using validated and objective measures) and reporting standards (including the use of magnitude of associations) [8, 15, 19, 33].

We did not identify any recent systematic reviews (within the past 5 years) that examined psychological factors, early healthcare use and motor dysfunctions. It would be helpful to undertake updated systematic reviews to explore the association of these factors with the prognosis of whiplash.

Our methodology had the benefit of relying on the best available evidence provided by the systematic reviews included, but this has limitations. More recent studies would not have been captured by the included reviews. In addition, by including all the prognostic factors explored by the systematic reviews, this meta-review maps the field and provides an overall picture, but in doing so, it necessarily reduces the depth of analysis for each individual factor.

In conclusion, this meta-review provides a comprehensive overview of the state of the high-level evidence available concerning the factors associated with the outcome of patients with whiplash injuries. The predictors of poor outcome after acute whiplash injury are early pain and some psychosocial factors, whereas physical factors are not associated with the outcome of acute whiplash.

## Electronic supplementary material

Below is the link to the electronic supplementary material.

Supplementary material 1 (DOC 63 kb)

Supplementary material 2 (DOCX 32 kb)

Supplementary material 3 (DOCX 203 kb)
