# Supplementary material for: Factors predicting outcome in whiplash injury: a systematic meta-review of prognostic factors
Source: J Orthop Traumatol. 2016 Oct 13;18(1):9–16. doi: 10.1007/s10195-016-0431-x (PMC5311004; doi:10.1007/s10195-016-0431-x)
Supplement: Supplementary file 2 — Supplementary material 2 (DOCX 32 kb) [file 10195_2016_431_MOESM2_ESM.docx]

Table 5. Search strategy and number of findings in various electronic data bases. Search was first undertaken at 14 Feb 2014, then updated at 14 May 15.

| **Database** | **Search strategy** | **Number of findings** |
| --- | --- | --- |
| Medline | 1. Whiplash Injuries/co, di, ep, et, hi, im, nu, pa, pp, pc, px, ra, ri, rt, rh, su, th, us [Complications, Diagnosis, Epidemiology, Etiology, History, Immunology, Nursing, Pathology, Physiopathology, Prevention & Control, Psychology, Radiography, Radionuclide Imaging, Radiotherapy, Rehabilitation, Surgery, Therapy, Ultrasonography] 2. limit 1 to (English language and systematic reviews) | 96 |
| Medline | 1. (whiplash and prognos*).af. 2. limit 5 to (english and systematic reviews) | 32 |
| Embase | 1. whiplash injury/co, di, dm, dt, ep, et, pc, rt, rh, si, su, th [Complication, Diagnosis, Disease Management, Drug Therapy, Epidemiology, Etiology, Prevention, Radiotherapy, Rehabilitation, Side Effect, Surgery, Therapy] 2. limit 1 to (English language and "systematic review" | 29 |
| Embase | 1. (whiplash and prognos*).af.  2. limit 5 to (english and systematic reviews) | 10 |
| Cochrane library | 1. MeSH descriptor: [Whiplash Injuries] with qualifier(s) Diagnosis; Epidemiology; Etiology; Pathology; Physiopathology; Prevention & control; Radiography; Radiotherapy; Rehabilitation; Therapy 2. Limit 1 to Cochrane reviews and other reviews | 24 |
| Cochrane library | 1. "whiplash" and "prognostic factor" 2. Limit 1 to Cochrane reviews and other reviews | 12 |
| CinAHL | 1. (MH "Whiplash Injuries/DI/DH/DT/ED/EP/ET/IM/ME/NU/PA/PP/PC/PR/PF/RH/RT/RA/RF/SU/SS/TH/TD/CO") 2. limit 1 to (English language and "systematic review") | 44 |
| CinAHL | 1. whiplash and prognostic factors  2. limit 5 to (english and systematic reviews) | 5 |
| PsycINFO | 1. exp Whiplash/ 2. limit 1 to (English language and "0830 systematic review") | 15 |
| PsycINFO | 1. (whiplash and prognos*).af.  2. limit 5 to (english and systematic reviews) | 43 |
| PubMed | (whiplash[MeSH Terms]) AND "systematic review" | 40 |
| PubMed | (((whiplash) AND "systematic review")) AND prognos* | 15 |
|  | Total 365  Duplicates 158  Items for screening by title and abstracts 207 | |
