# Supplementary material for: Factors predicting outcome in whiplash injury: a systematic meta-review of prognostic factors
Source: J Orthop Traumatol. 2016 Oct 13;18(1):9–16. doi: 10.1007/s10195-016-0431-x (PMC5311004; doi:10.1007/s10195-016-0431-x)
Supplement: Supplementary file 3 — Supplementary material 3 (DOCX 203 kb) [file 10195_2016_431_MOESM3_ESM.docx]

**Table 6. Citations excluded by full-text evaluation. For each paper, only one reason for exclusion is mentioned; even if the paper could have been excluded based on multiple reasons.**

| **Citation** | **Reason for exclusion** |
| --- | --- |
| (Murgatroyd et al., 2015) | Topic not related to prognostic factors of acute whiplash injury |
| (Prang et al., 2015) | Topic not related to prognostic factors of acute whiplash injury |
| (Häggman-Henrikson et al., 2014) | Topic not related to prognostic factors of acute whiplash injury |
| (Worsfold, 2014) | Method not a systematic review. |
| (Van Oosterwijck et al., 2013) | Topic not related to prognostic factors of acute whiplash injury |
| (Häggman-Henrikson et al., 2013) | Topic not related to prognostic factors of acute whiplash injury |
| (Stone et al., 2013) | Topic not related to prognostic factors of acute whiplash injury |
| (Kroeling et al., 2013) | Topic not related to prognostic factors of acute whiplash injury |
| (Walton et al., 2013) | This is an overview of systematic reviews, not a systematic review. This study is cited and discussed in the paper, but not analysed along with other systematic reviews. |
| (Silva and Cruz, 2013) | Topic not related to prognostic factors of acute whiplash injury |
| (Smith and Bolton, 2013) | Topic not related to prognostic factors of acute whiplash injury |
| (Michaleff and Ferreira, 2012) | Topic not related to prognostic factors of acute whiplash injury |
| (Gross et al., 2012) | Topic not related to prognostic factors of acute whiplash injury |
| (Carstensen, 2012) | Method not a systematic review |
| (Meeus et al., 2012) | Topic not related to prognostic factors of acute whiplash injury |
| (Watanabe et al., 2012) | Topic not related to prognostic factors of acute whiplash injury |
| (Laisne et al., 2012) | Topic not related to prognostic factors of acute whiplash injury |
| (Epstein and Klasser, 2011) | Topic not related to prognostic factors of acute whiplash injury |
| (Pastakia and Kumar, 2011) | Topic not related to prognostic factors of acute whiplash injury |
| (Zhang et al., 2011) | Topic not related to prognostic factors of acute whiplash injury |
| (Langevin et al., 2011) | Topic not related to prognostic factors of acute whiplash injury |
| (Goldsmith et al., 2011) | Topic not related to prognostic factors of acute whiplash injury |
| (Howell, 2011) | Topic not related to prognostic factors of acute whiplash injury |
| (Posadzki and Ernst, 2011) | Topic not related to prognostic factors of acute whiplash injury |
| (Axelsson and Marnetoft, 2010) | Topic not related to prognostic factors of acute whiplash injury |
| (van Hartingsveld et al., 2010) | Topic not related to prognostic factors of acute whiplash injury |
| (Shaw et al., 2010) | Topic not related to prognostic factors of acute whiplash injury |
| (Tullar et al., 2010) | Topic not related to prognostic factors of acute whiplash injury |
| (van Suijlekom et al., 2010) | Method not a systematic review |
| (Teasell et al., 2010a) | Topic not related to prognostic factors of acute whiplash injury |
| (Teasell et al., 2010b) | Topic not related to prognostic factors of acute whiplash injury |
| (Teasell et al., 2010c) | Topic not related to prognostic factors of acute whiplash injury |
| (Teasell et al., 2010d) | Topic not related to prognostic factors of acute whiplash injury |
| (Teasell et al., 2010e) | Topic not related to prognostic factors of acute whiplash injury |
| (Teasell et al., 2010f) | Topic not related to prognostic factors of acute whiplash injury |
| (Hoy et al., 2010) | Topic not related to prognostic factors of acute whiplash injury |
| (Walton, 2009) | Topic not related to prognostic factors of acute whiplash injury |
| (Lakke et al., 2009) | Topic not related to prognostic factors of acute whiplash injury |
| (Haneline, 2009) | Topic not related to prognostic factors of acute whiplash injury |
| (Haines et al., 2009) | Topic not related to prognostic factors of acute whiplash injury |
| (Fernandez et al., 2009) | Method not a systematic review |
| (Ernst, 2009) | Topic not related to prognostic factors of acute whiplash injury |
| (Scott et al., 2009) | Topic not related to prognostic factors of acute whiplash injury |
| (Hartman et al., 2009) | Topic not related to prognostic factors of acute whiplash injury |
| (Jansen et al., 2008) | Topic not related to prognostic factors of acute whiplash injury |
| (Wright et al., 2008) | Topic not related to prognostic factors of acute whiplash injury |
| (Holm et al., 2008) | Topic not related to prognostic factors of acute whiplash injury |
| (Cassidy and Côté, 2008) | Topic not related to prognostic factors of acute whiplash injury |
| (Drescher et al., 2008) | Topic not related to prognostic factors of acute whiplash injury |
| (Hurwitz et al., 2008) | Topic not related to prognostic factors of acute whiplash injury |
| (Nee, 2008) | Method not a systematic review |
| (Carroll et al., 2008) | Topic not related to prognostic factors of acute whiplash injury |
| (Poorbaugh et al., 2008) | Topic not related to prognostic factors of acute whiplash injury |
| (Hansson, 2007) | Topic not related to prognostic factors of acute whiplash injury |
| (Mercer et al., 2007) | Topic not related to prognostic factors of acute whiplash injury |
| (Gross et al., 2007) | Topic not related to prognostic factors of acute whiplash injury |
| (Schofferman et al., 2007) | Method not a systematic review |
| (Peloso et al., 2007) | Topic not related to prognostic factors of acute whiplash injury |
| (Jensen and Harms-Ringdahl, 2007) | Topic not related to prognostic factors of acute whiplash injury |
| (Verhagen Arianne et al., 2007) | Topic not related to prognostic factors of acute whiplash injury |
| (Lundmark and Persson, 2006) | Topic not related to prognostic factors of acute whiplash injury |
| (Peloso et al., 2006) | Topic not related to prognostic factors of acute whiplash injury |
| (Graham et al., 2006) | Topic not related to prognostic factors of acute whiplash injury |
| (Centeno et al., 2005) | Method not a systematic review |
| (Kroeling et al., 2005) | Topic not related to prognostic factors of acute whiplash injury |
| (Benoist, 2005) | Method not a systematic review |
| (Conlin et al., 2005a) | Topic not related to prognostic factors of acute whiplash injury |
| (Conlin et al., 2005b) | Topic not related to prognostic factors of acute whiplash injury |
| (Kay et al., 2005) | Topic not related to prognostic factors of acute whiplash injury |
| (Ameratunga et al., 2004) | Topic not related to prognostic factors of acute whiplash injury |
| (Griffiths et al., 2004) | Method not a systematic review |
| (Seferiadis et al., 2004) | Topic not related to prognostic factors of acute whiplash injury |
| (Freund and Schwartz, 2004) | Topic not related to prognostic factors of acute whiplash injury |
| (McLean and Clauw, 2004) | Method not a systematic review |
| (Kwan and Friel, 2003) | Method not a systematic review |
| (McClune et al., 2003) | Topic not related to prognostic factors of acute whiplash injury |
| (van Hout et al., 2003) | Topic not related to prognostic factors of acute whiplash injury |
| (Barnsley, 2003) | Topic not related to prognostic factors of acute whiplash injury |
| (Kwan and Fiel, 2002) | Topic not related to prognostic factors of acute whiplash injury |
| (McClune et al., 2002) | Topic not related to prognostic factors of acute whiplash injury |
| (Scholten-Peeters et al., 2002) | Topic not related to prognostic factors of acute whiplash injury |
| (Peeters et al., 2001) | Topic not related to prognostic factors of acute whiplash injury |
| (Young, 2001) | Topic not related to prognostic factors of acute whiplash injury |
| (Kessels et al., 2000) | Topic not related to prognostic factors of acute whiplash injury |
| (Magee et al., 2000) | Topic not related to prognostic factors of acute whiplash injury |
| (Bogduk, 2000) | Topic not related to prognostic factors of acute whiplash injury. |
| (Provinciali and Baroni, 1999) | Method not a systematic review |
| (Freeman et al., 1999) | Topic not related to prognostic factors of acute whiplash injury |
| (Richell-Herren, 1999) | Topic not related to prognostic factors of acute whiplash injury |
| (Bogduk and Lord, 1998) | Method not a systematic review |

AMERATUNGA, S. N., NORTON, R. N., BENNETT, D. A. & JACKSON, R. T. 2004. Risk of disability due to car crashes: a review of the literature and methodological issues. *Injury,* 35**,** 1116-27.

AXELSSON, I. & MARNETOFT, S. 2010. Benefits and harms of sick leave: lack of randomized, controlled trials. *International Journal of Rehabilitation Research,* 33**,** 1-3.

BARNSLEY, L. 2003. An evidence-based approach to the treatment of acute whiplash injury. *Pain Research & Management,* 8**,** 33-6.

BENOIST, M. 2005. The Michel Benoist and Robert Mulholland yearly European Spine Journal Review: A survey of the "medical" articles in the European Spine Journal, 2004. *European Spine Journal,* 14**,** 3-8.

BOGDUK, N. 2000. Whiplash: "why pay for what does not work?"... World Congress on Whiplash-Associated Disorders in Vancouver, British Columbia, Canada in February of 1999. *Journal of Musculoskeletal Pain,* 8**,** 29-53.

BOGDUK, N. & LORD, S. M. 1998. Cervical spine disorders. *Current Opinion in Rheumatology,* 10**,** 110-5.

CARROLL, L. J., HURWITZ, E. L., COTE, P., HOGG-JOHNSON, S., CARRAGEE, E. J., NORDIN, M., HOLM, L. W., VAN DER VELDE, G., CASSIDY, J. D., GUZMAN, J., PELOSO, P. M., HALDEMAN, S., BONE, JOINT DECADE - TASK FORCE ON NECK, P. & ITS ASSOCIATED, D. 2008. Research priorities and methodological implications: the Bone and Joint Decade 2000-2010 Task Force on Neck Pain and Its Associated Disorders. *Spine,* 33**,** S214-20.

CARSTENSEN, T. B. 2012. The influence of psychosocial factors on recovery following acute whiplash trauma. *Danish Medical Journal,* 59**,** B4560.

CASSIDY, J. D. & CÔTÉ, P. 2008. Is it time for a population health approach to neck pain? *Journal of Manipulative & Physiological Therapeutics,* 31**,** 442-446.

CENTENO, C. J., FREEMAN, M. & ELKINS, W. L. 2005. A review of the literature refuting the concept of minor impact soft tissue injury. *Pain Research & Management,* 10**,** 71-4.

CONLIN, A., BHOGAL, S., SEQUEIRA, K. & TEASELL, R. 2005a. Treatment of whiplash-associated disorders--part I: Non-invasive interventions. *Pain Research & Management,* 10**,** 21-32.

CONLIN, A., BHOGAL, S., SEQUEIRA, K. & TEASELL, R. 2005b. Treatment of whiplash-associated disorders - part II: medical and surgical interventions (Structured abstract). *Pain Research and Management* [Online], 10. Available: <http://onlinelibrary.wiley.com/o/cochrane/cldare/articles/DARE-12005000022/frame.html>.

DRESCHER, K., HARDY, S., MACLEAN, J., SCHINDLER, M., SCOTT, K. & HARRIS, S. R. 2008. Efficacy of postural and neck-stabilization exercises for persons with acute whiplash-associated disorders: a systematic review. *Physiotherapy Canada,* 60**,** 215-223.

EPSTEIN, J. B. & KLASSER, G. D. 2011. Whiplash-associated disorders and temporomandibular symptoms following motor-vehicle collisions. *Quintessence International,* 42**,** e1-e14.

ERNST, E. 2009. Acupuncture: What Does the Most Reliable Evidence Tell Us? *Journal of Pain and Symptom Management,* 37**,** 709-714.

FERNANDEZ, C. E., AMIRI, A., JAIME, J. & DELANEY, P. 2009. The relationship of whiplash injury and temporomandibular disorders: a narrative literature review. *Journal of Chiropractic Medicine,* 8**,** 171-186.

FREEMAN, M. D., CROFT, A. C., ROSSIGNOL, A. M., WEAVER, D. S. & REISER, M. 1999. A review and methodologic critique of the literature refuting whiplash syndrome. *Spine,* 24**,** 86-96.

FREUND, B. & SCHWARTZ, M. 2004. Whiplash associated disorders: A review of recent advances in treatment. *Journal of Whiplash and Related Disorders,* 3**,** 49-58.

GOLDSMITH, C. H., GROSS, A. R., MACDERMID, J., SANTAGUIDA, P. L. & MILLER, J. 2011. What does the evidence tell us about design of future treatment trials for whiplash-associated disorders? *Spine,* 36**,** S292-302.

GRAHAM, N., GROSS, A. R., GOLDSMITH, C. & CERVICAL OVERVIEW, G. 2006. Mechanical traction for mechanical neck disorders: a systematic review. *Journal of Rehabilitation Medicine,* 38**,** 145-52.

GRIFFITHS, H. J., KIDWAI, A. S. & WRIGHT, W. C. 2004. Hyperextension strain or whiplash injuries to the cervical spine - Revisited. *Journal of Whiplash and Related Disorders,* 3**,** 25-45.

GROSS, A., FORGET, M., ST GEORGE, K., FRASER MICHELLE, M. H., GRAHAM, N., PERRY, L., BURNIE STEPHEN, J., GOLDSMITH CHARLES, H., HAINES, T. & BRUNARSKI, D. 2012. Patient education for neck pain. *Cochrane Database of Systematic Reviews* [Online]. Available: <http://onlinelibrary.wiley.com/doi/10.1002/14651858.CD005106.pub4/abstract>.

GROSS, A. R., GOLDSMITH, C., HOVING, J. L., HAINES, T., PELOSO, P., AKER, P., SANTAGUIDA, P. & MYERS, C. 2007. Conservative management of mechanical neck disorders: a systematic review. *Journal of Rheumatology,* 34**,** 1083-1102.

HÄGGMAN-HENRIKSON, B., LIST, T., WESTERGREN, H. & AXELSSON, S. 2013. Temporomandibular Disorder Pain After Whiplash Trauma: A Systematic Review. *Journal of Orofacial Pain,* 27**,** 217-226.

HÄGGMAN-HENRIKSON, B., REZVANI, M. & LIST, T. 2014. Prevalence of whiplash trauma in TMD patients: a systematic review. *Journal of Oral Rehabilitation,* 41**,** 59-68.

HAINES, T., GROSS, A., BURNIE, S. J., GOLDSMITH, C. H. & PERRY, L. 2009. Patient education for neck pain with or without radiculopathy. *Cochrane Database of Systematic Reviews***,** CD005106.

HANELINE, M. T. 2009. The notion of a "whiplash culture": a review of the evidence. *Journal of Chiropractic Medicine,* 8**,** 119-124.

HANSSON, E. E. 2007. Vestibular rehabilitation - for whom and how? A systematic review. *Advances in Physiotherapy,* 9**,** 106-116.

HARTMAN, T. C., BORGHUIS, M. S., LUCASSEN, P. L., VAN DE LAAR, F. A., SPECKENS, A. E. & VAN WEEL, C. 2009. Medically unexplained symptoms, somatisation disorder and hypochondriasis: Course and prognosis. A systematic review. *Journal of Psychosomatic Research,* 66**,** 363-377.

HOLM, L. W., CARROLL, L. J., CASSIDY, J. D., HOGG-JOHNSON, S., CÔTÉ, P., GUZMAN, J., PELOSO, P., NORDIN, M., HURWITZ, E., CARRAGEE, E. & HALDEMAN, S. 2008. The burden and determinants of neck pain in whiplash-associated disorders after traffic collisions: results of the Bone and Joint Decade 2000-2010 Task Force on Neck Pain and Its Associated Disorders. *Spine,* 33**,** S52-9.

HOWELL, E. R. 2011. The association between neck pain, the Neck Disability Index and cervical ranges of motion: a narrative review. *Journal of the Canadian Chiropractic Association,* 55**,** 211-221.

HOY, D. G., PROTANI, M., DE, R. & BUCHBINDER, R. 2010. The epidemiology of neck pain. *Best Practice and Research: Clinical Rheumatology,* 24**,** 783-792.

HURWITZ, E. L., CARRAGEE, E. J., CARROLL, L. J., NORDIN, M., GUZMAN, J., PELOSO, P. M., HOLM, L. W., CÔTÉ, P., HOGG-JOHNSON, S., CASSIDY, J. D. & HALDEMAN, S. 2008. Treatment of neck pain: noninvasive interventions: results of the Bone and Joint Decade 2000-2010 Task Force on Neck Pain and Its Associated Disorders. *Spine,* 33**,** S123-52.

JANSEN, G. B., EDLUND, C., GRANE, P., HILDINGSSON, C., KARLBERG, M., LINK, H., MAWE, U., PORTALA, K., RYDEVIK, B., STERNER, Y., SWEDISH SOCIETY OF, M. & WHIPLASH COMMISSION MEDICAL TASK, F. 2008. Whiplash injuries: diagnosis and early management. The Swedish Society of Medicine and the Whiplash Commission Medical Task Force. *European Spine Journal,* 17 Suppl 3**,** S355-417.

JENSEN, I. & HARMS-RINGDAHL, K. 2007. Strategies for prevention and management of musculoskeletal conditions. Neck pain. *Best Practice & Research in Clinical Rheumatology,* 21**,** 93-108.

KAY, T. M., GROSS, A., GOLDSMITH, C., SANTAGUIDA, P. L., HOVING, J., BRONFORT, G. & CERVICAL OVERVIEW, G. 2005. Exercises for mechanical neck disorders. *Cochrane Database of Systematic Reviews***,** CD004250.

KESSELS, R. P., ALEMAN, A., VERHAGEN, W. I. & VAN LUIJTELAAR, E. L. 2000. Cognitive functioning after whiplash injury: a meta-analysis. *Journal of the International Neuropsychological Society,* 6**,** 271-8.

KROELING, P., GROSS, A., GRAHAM, N., BURNIE, S. J., SZETO, G., GOLDSMITH, C. H., HAINES, T. & FORGET, M. 2013. Electrotherapy for neck pain. *Cochrane Database of Systematic Reviews,* 8**,** CD004251.

KROELING, P., GROSS, A. R. & GOLDSMITH, C. H. 2005. A Cochrane review of electrotherapy for mechanical neck disorders. *Spine,* 30**,** E641-8.

KWAN, O. & FIEL, J. 2002. Critical appraisal of facet joints injections for chronic whiplash. *Medical Science Monitor,* 8**,** RA191-5.

KWAN, O. & FRIEL, J. 2003. A review and methodologic critique of the literature supporting 'chronic whiplash injury': part I - research articles. *Medical Science Monitor,* 9**,** RA203-15.

LAISNE, F., LECOMTE, C. & CORBIERE, M. 2012. Biopsychosocial predictors of prognosis in musculoskeletal disorders: A systematic review of the literature (corrected and republished)*. *Disability and Rehabilitation: An International, Multidisciplinary Journal,* 34**,** 1912-1941.

LAKKE, S. E., SOER, R., TAKKEN, T. & RENEMAN, M. F. 2009. Risk and prognostic factors for non-specific musculoskeletal pain: a synthesis of evidence from systematic reviews classified into ICF dimensions. *Pain,* 147**,** 153-64.

LANGEVIN, P., LOWCOCK, J., WEBER, J., NOLAN, M., GROSS, A. R., PELOSO, P. M., ROBERTS, J., GRAHAM, N., GOLDSMITH, C. H., BURNIE, S. J. & HAINES, T. 2011. Botulinum toxin intramuscular injections for neck pain: A systematic review and metaanalysis. *Journal of Rheumatology,* 38**,** 203-214.

LUNDMARK, H. & PERSSON, A. L. 2006. Physiotherapy and management in early whiplash-associated disorders (WAD): a review (Provisional abstract). *Advances in Physiotherapy* [Online], 8. Available: <http://onlinelibrary.wiley.com/o/cochrane/cldare/articles/DARE-12007009096/frame.html>.

MAGEE, D. J., OBORN-BARRETT, E., TURNER, S. & FENNING, N. 2000. A systematic overview of the effectiveness of physical therapy intervention on soft tissue neck injury following trauma (Structured abstract). *Physiotherapy Canada* [Online], 52. Available: <http://onlinelibrary.wiley.com/o/cochrane/cldare/articles/DARE-12000005194/frame.html>.

MCCLUNE, T., BURTON, A. K. & WADDELL, G. 2002. Whiplash associated disorders: a review of the literature to guide patient information and advice. *Emergency Medicine Journal,* 19**,** 499-506.

MCCLUNE, T., BURTON, A. K. & WADDELL, G. 2003. Evaluation of an evidence based patient educational booklet for management of whiplash associated disorders. *Emergency Medicine Journal,* 20**,** 514-7.

MCLEAN, S. A. & CLAUW, D. J. 2004. Predicting chronic symptoms after an acute "stressor"--lessons learned from 3 medical conditions. *Medical Hypotheses,* 63**,** 653-8.

MEEUS, M., NIJS, J., HAMERS, V., ICKMANS, K. & VAN OOSTERWIJCKS, J. 2012. The efficacy of patient education in whiplash associated disorders: A systematic review. *Pain Physician,* 15**,** 351-361.

MERCER, C., JACKSON, A. & MOORE, A. 2007. Developing clinical guidelines for the physiotherapy management of whiplash associated disorder (WAD). *International Journal of Osteopathic Medicine,* 10**,** 50-54.

MICHALEFF, Z. A. & FERREIRA, M. L. 2012. Physiotherapy rehabilitation for whiplash associated disorder II: a systematic review and meta-analysis of randomised controlled trials. *British Journal of Sports Medicine,* 46**,** 662-663.

MURGATROYD, D. F., CASEY, P. P., CAMERON, I. D. & HARRIS, I. A. 2015. The effect of financial compensation on health outcomes following musculoskeletal injury: Systematic review. *PLoS ONE,* 10.

NEE, P. A. 2008. Influence of a previous neck sprain on recovery after whiplash injury. *Injury,* 39**,** 1442-3.

PASTAKIA, K. & KUMAR, S. 2011. Acute whiplash associated disorders (WAD). *Open Access Emergency Medicine,* 3**,** 29-32.

PEETERS, G. G., VERHAGEN, A. P., DE BIE, R. A. & OOSTENDORP, R. A. 2001. The efficacy of conservative treatment in patients with whiplash injury: a systematic review of clinical trials. *Spine,* 26**,** E64-73.

PELOSO, P. M., GROSS, A., HAINES, T., TRINH, K., GOLDSMITH, C. H. & BURNIE, S. 2007. Medicinal and injection therapies for mechanical neck disorders. *Cochrane Database of Systematic Reviews,* (3).

PELOSO, P. M., GROSS, A. R., HAINES, T. A., TRINH, K., GOLDSMITH, C. H. & AKER, P. 2006. Medicinal and injection therapies for mechanical neck disorders: a Cochrane systematic review. *Journal of Rheumatology,* 33**,** 957-967.

POORBAUGH, K., BRISMEE, J. M., PHELPS, V. & SIZER, P. S., JR. 2008. Late whiplash syndrome: a clinical science approach to evidence-based diagnosis and management. *Pain Practice,* 8**,** 65-87; quiz 88-9.

POSADZKI, P. & ERNST, E. 2011. Spinal manipulation: An update of a systematic review of systematic reviews. *New Zealand Medical Journal,* 124**,** 9.

PRANG, K.-H., NEWNAM, S. & BERECKI-GISOLF, J. 2015. The impact of family and work-related social support on musculoskeletal injury outcomes: A systematic review. *Journal of Occupational Rehabilitation,* 25**,** 207-219.

PROVINCIALI, L. & BARONI, M. 1999. Clinical approaches to whiplash injuries: a review. *Critical Reviews in Physical & Rehabilitation Medicine,* 11**,** 339-368.

RICHELL-HERREN, K. 1999. Towards evidence based emergency medicine: best BETS from the Manchester Royal Infirmary. Mobilisation of neck sprains. *Journal of Accident & Emergency Medicine,* 16**,** 363.

SCHOFFERMAN, J., BOGDUK, N. & SLOSAR, P. 2007. Chronic whiplash and whiplash-associated disorders: an evidence-based approach. *Journal of the American Academy of Orthopaedic Surgeons,* 15**,** 596-606.

SCHOLTEN-PEETERS, G. G., BEKKERING, G. E., VERHAGEN, A. P., VAN DER WINDT, D. A., LANSER, K., HENDRIKS, E. J. & OOSTENDORP, R. A. 2002. Clinical practice guideline for the physiotherapy of patients with whiplash-associated disorders. *Spine,* 27**,** 412-22.

SCOTT, N. A., GUO, B., BARTON, P. M. & GERWIN, R. D. 2009. Trigger point injections for chronic non-malignant musculoskeletal pain: a systematic review. *Pain Medicine,* 10**,** 54-69.

SEFERIADIS, A., ROSENFELD, M. & GUNNARSSON, R. 2004. A review of treatment interventions in whiplash-associated disorders. *European Spine Journal,* 13**,** 387-97.

SHAW, L., DESCARREAUX, M., BRYANS, R., DURANLEAU, M., MARCOUX, H., POTTER, B., RUEGG, R., WATKIN, R. & WHITE, E. 2010. A systematic review of chiropractic management of adults with whiplash-associated disorders: Recommendations for advancing evidence-based practice and research. *Work: Journal of Prevention, Assessment & Rehabilitation,* 35**,** 369-394.

SILVA, A. G. & CRUZ, A. L. 2013. Standing balance in patients with whiplash-associated neck pain and idiopathic neck pain when compared with asymptomatic participants: A systematic review. *Physiotherapy Theory & Practice,* 29**,** 1-18.

SMITH, J. & BOLTON, P. S. 2013. What Are the Clinical Criteria Justifying Spinal Manipulative Therapy for Neck Pain?- A Systematic Review of Randomized Controlled Trials. *Pain Medicine (United States),* 14**,** 460-468.

STONE, A. M., VICENZINO, B., LIM, E. C. W. & STERLING, M. 2013. Measures of central hyperexcitability in chronic whiplash associated disorder - A systematic review and meta-analysis. *Manual Therapy,* 18**,** 111-117.

TEASELL, R. W., MCCLURE, J. & WALTON, D. 2010a. Toward an evidence-based approach to whiplash injuries. *Pain Research & Management,* 15**,** 285-286.

TEASELL, R. W., MCCLURE, J., WALTON, D., PRETTY, J., SALTER, K., MEYER, M., SEQUEIRA, K. & DEATH, B. 2010b. A research synthesis of therapeutic interventions for whiplash-associated disorder (WAD): Part 3-Interventions for subacute WAD. *Pain Research & Management,* 15**,** 305-312.

TEASELL, R. W., MCCLURE, J., WALTON, D., PRETTY, J., SALTER, K., MEYER, M., SEQUEIRA, K. & DEATH, B. 2010c. A research synthesis of therapeutic interventions for whiplash-associated disorder (WAD): Part 4-Noninvasive interventions for chronic WAD. *Pain Research & Management,* 15**,** 313-322.

TEASELL, R. W., MCCLURE, J., WALTON, D., PRETTY, J., SALTER, K., MEYER, M., SEQUEIRA, K. & DEATH, B. 2010d. A research synthesis of therapeutic interventions for whiplash-associated disorder (WAD): Part 5-Surgical and injection-based interventions for chronic WAD. *Pain Research & Management,* 15**,** 323-334.

TEASELL, R. W., MCCLURE, J. A., WALTON, D., PRETTY, J., SALTER, K., MEYER, M., SEQUEIRA, K. & DEATH, B. 2010e. A research synthesis of therapeutic interventions for whiplash-associated disorder (WAD): Part 2 - Interventions for acute WAD. *Pain Research and Management,* 15**,** 295-304.

TEASELL, R. W., MCCLURE, J. A., WALTON, D., PRETTY, J., SALTER, K., MEYER, M., SEQUEIRA, K. & DEATH, B. 2010f. A research synthesis of therapeutic interventions for whiplash-associated disorder. Part 1: overview and summary (Provisional abstract). *Pain Research and Management* [Online], 15. Available: <http://onlinelibrary.wiley.com/o/cochrane/cldare/articles/DARE-12010008084/frame.html>.

TULLAR, J. M., BREWER, S., AMICK, B. C., III, IRVIN, E., MAHOOD, Q., POMPEII, L. A., WANG, A., VAN EERD, D., GIMENO, D. & EVANOFF, B. 2010. Occupational safety and health interventions to reduce musculoskeletal symptoms in the health care sector. *Journal of Occupational Rehabilitation,* 20**,** 199-219.

VAN HARTINGSVELD, F., OSTELO, R. W., CUIJPERS, P., DE VOS, R., RIPHAGEN, I. I. & DE VET, H. C. 2010. Treatment-related and patient-related expectations of patients with musculoskeletal disorders: A systematic review of published measurement tools. *The Clinical Journal of Pain,* 26**,** 470-488.

VAN HOUT, M. S., WEKKING, E. M., BERG, I. J. & DEELMAN, B. G. 2003. Psychological treatment of patients with chronic toxic encephalopathy: lessons from studies of chronic fatigue and whiplash. *Psychotherapy & Psychosomatics,* 72**,** 235-44.

VAN OOSTERWIJCK, J., NIJS, J., MEEUS, M. & PAUL, L. 2013. Evidence for central sensitization in chronic whiplash: A systematic literature review. *European Journal of Pain,* 17**,** 299-312.

VAN SUIJLEKOM, H., MEKHAIL, N., PATEL, N., VAN ZUNDERT, J., VAN KLEEF, M. & PATIJN, J. 2010. 7. Whiplash-associated disorders. *Pain Practice,* 10**,** 131-6.

VERHAGEN ARIANNE, P., SCHOLTEN-PEETERS GWENDOLIJNE, G. G. M., VAN WIJNGAARDEN, S., DE BIE, R. & BIERMA-ZEINSTRA SITA, M. A. 2007. Conservative treatments for whiplash. *Cochrane Database of Systematic Reviews* [Online]. Available: <http://onlinelibrary.wiley.com/doi/10.1002/14651858.CD003338.pub3/abstract>.

WALTON, D. 2009. A review of the definitions of 'recovery' used in prognostic studies on whiplash using an ICF framework. *Disability and Rehabilitation,* 31**,** 943-957.

WALTON, D. M., CARROLL, L. J., KASCH, H., STERLING, M., VERHAGEN, A. P., MACDERMID, J. C., GROSS, A., SANTAGUIDA, P. L. & CARLESSO, L. 2013. An Overview of Systematic Reviews on Prognostic Factors in Neck Pain: Results from the International Collaboration on Neck Pain (ICON) Project. *Open Orthop J,* 7**,** 494-505.

WATANABE, T. K., BELL, K. R., WALKER, W. C. & SCHOMER, K. 2012. Systematic review of interventions for post-traumatic headache. *Pm & R,* 4**,** 129-40.

WORSFOLD, C. 2014. When range of motion is not enough: towards an evidence-based approach to medico-legal reporting in whiplash injury. *Journal of Forensic & Legal Medicine,* 25**,** 95-9.

WRIGHT, C., RUSHTON, A., GAMLIN, J. & FARRELL, D. 2008. Systematic reviews in clinical practice: evaluating evidence for management of acute whiplash-associated disorder. *International Journal of Therapy & Rehabilitation,* 15**,** 333-342.

YOUNG, W. F. 2001. The enigma of whiplash injury. Current management strategies and controversies. *Postgraduate Medicine,* 109**,** 179-80, 183-6.

ZHANG, T., ADATIA, A., ZARIN, W., MOITRI, M., VIJENTHIRA, A., CHU, R., THABANE, L. & KEAN, W. 2011. The efficacy of botulinum toxin type A in managing chronic musculoskeletal pain: A systematic review and meta analysis. *Inflammopharmacology,* 19**,** 21-34.
